# Closure of Iterative Laparotomy in Patients With Previous Mesh Reinforcement a Cohorts’ Study. Short-Term Results

**DOI:** 10.3389/jaws.2022.10030

**Published:** 2022-03-23

**Authors:** A. Bravo-Salva, J.J. Sancho-Insenser, M. Pera-Román, J.A. Pereira-Rodriguez

**Affiliations:** ^1^ General and Digestive Surgery Department, Parc de Salut Mar, Hospital del Mar, Barcelona, Spain; ^2^ Department of Experimental and Health Science, Pompeu Fabra University, Barcelona, Spain; ^3^ Department of Surgery, Autonomous University of Barcelona, Hospital del Mar, Barcelona, Spain

**Keywords:** iterative laparotomy, prophylactic mesh, incisional hernia, abdominal wall reinforcement, incisional hernia prevention

## Abstract

**Purpose**: Due to extension of prophylactic mesh indications use will become more common to find patients receiving an iterative laparotomy (IL) over a previous reinforced abdominal wall. The aim of this study was to analyze outcomes after IL in patients with previous mesh reinforcement.

**Methods:** This study was a prospective secondary analysis of midline laparotomy closure performed from July 2017 to July 2018 registered in PHACPA study (NCT 02658955). IL were included and surgery characteristics and outcomes analyzed. We compared two groups: with (PreM) or without previous prophylactic onlay mesh reinforcement (PreS) Subgroups’ analysis, risk factors for complications and survival free hernia analysis were performed.

**Results:** 121 IL were analyzed. Only obesity was associated with higher SSO (OR 2.6; CI 95% 1.02–6.90; *p* = 0.04) There were 15 incisional hernias (IH) (14.4%). Group with previous mesh reinforcement (pre M) had a higher statistically significative incidence of IH (OR = 1.21; CI 95% 1.05–1.39; *p* = 0.015). Use of slowly absorbable suture (OR = 0.74; CI 95% 0.60–0.91; *p* = 0.001), USP 2/0 suture (OR, 0.31; 95% CI, 0.10–0.94; *p* = 0.033), and *small bites* technique (OR = 0.81; CI 95% 0.72–0.90; *p* = 0.011) were associated with less IH.

**Conclusion**: IL has a high percentage of complications and IH. In case of IL without previous reinforcement, a mesh can help to reduce IH. Our data cannot clearly support any technique to close an IL with previous mesh.

## Introduction

Incisional hernia (IH) after elective and emergency midline laparotomy can occur in up to 40% of high-risk patients [1, 2]. To prevent IH, several measures have been introduced, such as the “*small bites*“ (SB) technique, which has demonstrated a reduction in IH after elective surgery [3], and prophylactic mesh reinforcement in high-risk patients. [4, 5] Both techniques have been suggested by the European Hernia Society (EHS) guidelines [6].

Although previous laparotomy is a well-known risk factor for IH [2], it does not appear regularly in all studies on IH risk factors [7]. With the widespread use of prophylactic meshes [8], it is becoming more common to perform iterative laparotomy (IL) over a previously mesh-reinforced abdominal wall. To our knowledge, there are no data as to whether and how this situation influences the results of an IL. Moreover, it is unclear what would be the optimal way to close the abdominal wall and whether a new mesh could be effective in this scenario.

Thus, there are many questions when performing an IL with previous mesh reinforcement: Is there a risk of complications or IH increased? How should the abdominal wall be closed? What is the best suture material to close the abdominal wall with a previous mesh?

The aim of this study was to analyse the outcomes after IL in patients with previous mesh reinforcement and compare the different techniques of closure. As a control group, we used a series of patients with IL without previous mesh.

## Methods

This was an observational study consisting of a secondary analysis of patients from a prospective study of the implementation of SB [3] technique and prophylactic onlay mesh closure protocol after laparotomy in a General University Hospital (PHACPA study) [9]. The STROBE checklist was followed to ensure accurate reporting of observational analysis [10].

The aim of this study was to analyze the outcomes after IL in patients with previous mesh reinforcement.

Data on all patients that underwent a midline laparotomy (elective and emergency) during the period from July 2017 to July 2018 were collected. Information on patients with a previous midline laparotomy both with and without previous prophylactic onlay mesh was compiled. Compilation was carried separately for this study. All of them older than 18 years old were eligible to participate. Exclusion criteria included concomitant IH, death within the first 24 postoperative hours, open abdomen with progressive closure, re-laparotomy as a result of a complication of a recently performed operation (same episode iterative laparotomy (IL) or prior to the first postoperative month), and lack of patient’s data.

Data collected prospectively in the database included patients’ characteristics and comorbidities: sex, age, ASA score, body mass index, obesity, diabetes mellitus, heart disease, chronic obstructive pulmonary disease, liver disease, chronic renal failure, cancer history, smoking or immunosuppression treatment or disease; characteristics of previous operations: Surgery subtype: hepatopancreatobiliary surgery, gastrointestinal surgery, colorectal surgery or others; emergency or elective surgery. Diagnostic findings: surgery contamination, technical details of surgery: use of “small bites technique”, material of closure: use of long-term absorbable suture, USP 1/0 or 2/0 suture Caliber, whether a new prophylactic mesh was used or not, postoperative complications classified in accordance with Clavien-Dindo grades [11]: surgical site occurrences (SSO) such as surgical site infection (SSI), haematoma, wound seroma; need for reoperation, readmission, and follow-up duration [9]^.^

For the analysis of outcomes, two groups were compared: IL with previous prophylactic onlay mesh (PreM group) and IL without previous mesh (PreS group). Suture technique and material were included in analysis as well as use of a new prophylactic onlay mesh, new mesh positioning was performed after laparotomy closure, a 3 cm dissection on each side of the incision, a PVDF (DynaMeshÒ-CICAT, FEG Textiltechnik MbH) was used in the onlay position and sutured with polypropylene 2/0 stitches (Prolene, Ethicon) as explained in previous PHACPA study publication Two subgroups emerged from the analysis on each arm depending on the use a new mesh: previous mesh closed with suture (PreMs), previous mesh receiving a new mesh (PreMm), previous suture closed with a reinforcement onlay mesh (PreSm), and previous suture closed with a new suture (PreSs).

All of the patients were included in the analysis of postoperative outcomes. Follow up was performed by a surgeon in outpatient appointment with physical exploration at 1 month, 6 months, 1 year, and 2 years. In case of doubt, abdominal ultrasound or computed tomography was performed and IH was considered as any abdominal wall gap with or without a bulge in the area of a postoperative scar perceptible or palpable by clinical examination or imaging. The analysis of IH was performed only in patients with at least 6 months follow-up. A comparative risk for IH analysis between groups was performed (Figure 2) and a Cox regression survival analysis was performed to compare PreS and PreM groups hernia-free time (Figure 3).

The study was approved by the Clinical Research Ethics Committee (CREC number 2016/6543/I). The patients signed informed consent, and all of the data were processed in accordance with the Law 15/1999 on the Protection of Personal Data. The PAHCPA protocol was registered with the NCT02658955 identifier (Clinical Trials.gov).

Data analyses were performed using SPSS® statistical software, version 24.0 (IBM, Armonk, New York, United States). Comparative univariate and multivariate analyses were performed. Numeric variables were presented as mean and standard deviation (SD) or median and interquartile range, and categorical variables were reported as proportions. The association between qualitative variables was assessed using contingency tables (chi square test and Fisher’s exact test when necessary), and the quantitative tests were conducted using Student’s *t* test for unpaired data or the Mann–Whitney *U* test when necessary. The normality of the distribution of numeric variables was assessed using normal QQ plots. *p* < 0.05 was considered to be statistically significant. A Cox proportional hazards regression model was used to detect risk factors related to IH and a Cox free hernia survival curve was obtained for each study’s group.

## Results

**FIGURE 1:**
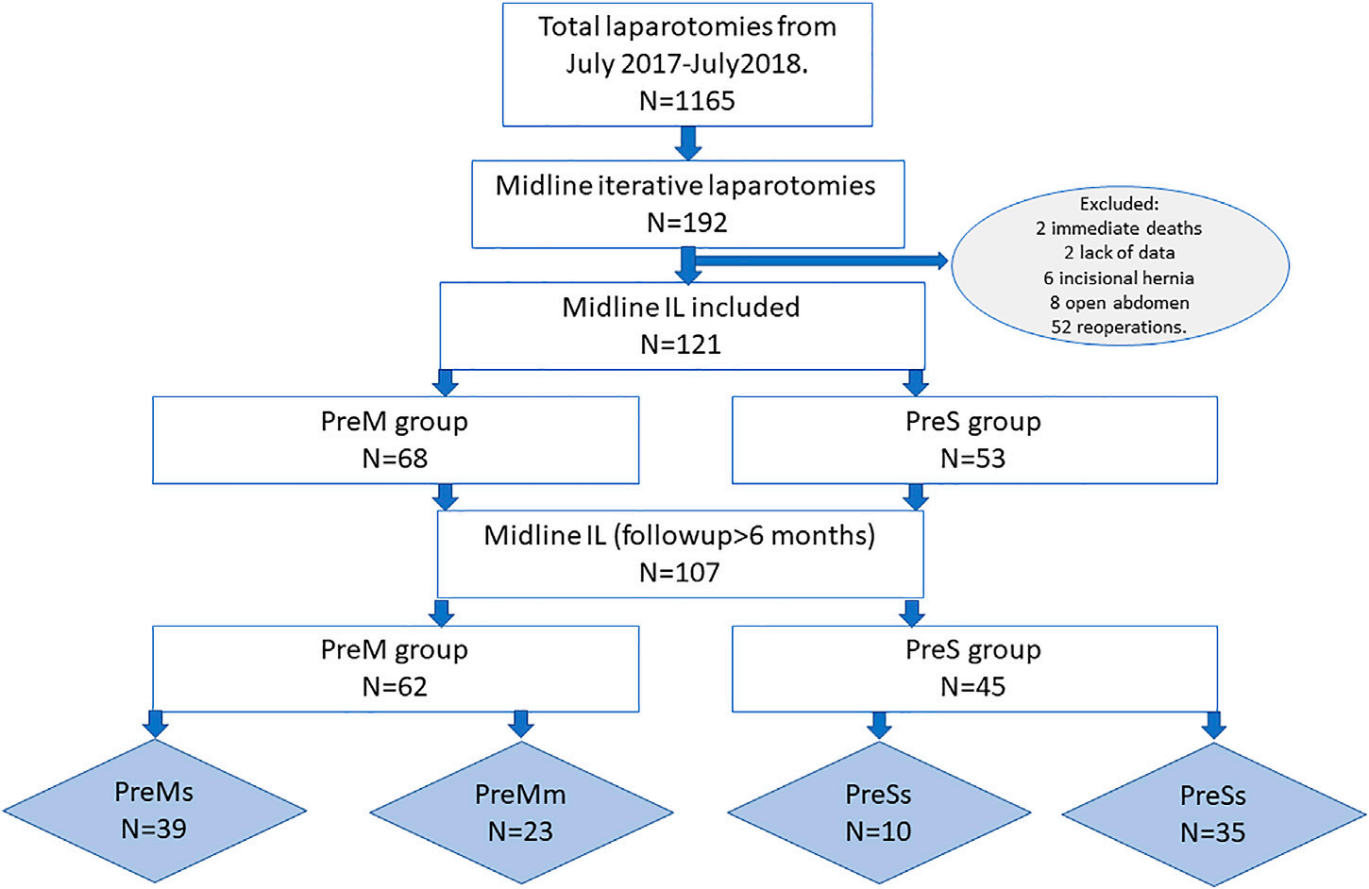
Study’s flowchart.

During the study period, a total of 1,165 laparotomies were performed, 192 of which were midline IL. A total of 121 IL were included for analysis. Causes of exclusion and the distribution of groups and subgroups are illustrated in Figure 1.

Table 1 shows and compares the demographic characteristics, associated comorbidities, and surgical details between the groups. All previous reinforcement meshes were non absorbable synthetic meshes. The groups were comparable in demographic characteristics and comorbidities, while some technical details were significantly different. Namely, SB technique (43.4% *vs.* 16.2%; *p* = 0.01), slowly absorbable suture (98.1% *vs*. 19.5% *p* = 0.01), and prophylactic mesh (79.2 vs. 36.8%; *p* = 0.01) were more frequently used in the PreS group.

**TABLE 1:** Patients’ and surgery characteristics.

N = 121	PreS n = 53	PreM n = 68	*p* value
Sex: female; n (%)	24 (45.3)	30 (44.1)	0.52
Age; mean Yr (SD)	68.4 (15)	70.2 (12)	0.47
Elderly >70years; n (%)	32 (45.7)	38 (54.3)	0.37
ASA III-IV; n (%)	36 (41,4)	52 (58.6)	0.25
IMC	25.2 (4.6)	26.8 (4.6)	0.06
Incisional hernia risk factors			
Obesity BMI > 30; n (%)	7 (13.2)	16 (23.5)	0.11
DM; n (%)	9 (17)	16 (23.5)	0.37
Heart disease; n (%)	10 (18.9)	15 (22.1)	0.61
COPD; n (%)	15 (28.3)	19 (27.9)	0.56
Liver disease; n (%)	6 (11.3)	3 (4.4)	0.13
CRF; n (%)	3 (5.7)	5 (7.4)	0.71
Neoplasm; n (%)	40 (75.5)	51 (75)	0.95
Smoking; n (%)	13 (24.5)	16 (23.5)	0.53
Immunosuppression; n (%)	3 (5.7)	3 (4.4)	0.52
Surgery characteristics			
Emergency surgery; n (%)	24 (45.3)	17 (25)	0.02
Type of suture; n (%)			0.001
Unknown	1 (1.9)	2 (2.9)	
Fast absorbable	2 (3.8)	0	
Slowly absorbable	49 (92.5)	23 (33.8)	
No absorbable	1 (1.9)	43 (63.2)	
Small bites; n (%)	23 (43.4)	11 (16.2)	0.01
Prophylactic mesh; n (%)	42 (79.2)	25 (36.8)	0.01
Surgery length; mean minutes (SD)	93 (66)	111 (64)	0.92

Yr, year; n, number; BMI, body mass index; DM, diabetes mellitus; COPD, chronic obstructive pulmonary disease; CRF, chronic renal failure; SD, standard deviation.

When comparing short-term outcomes (Table 2), there were no inter-group differences in overall complications and surgical site occurrence (SSO), but both groups showed a high rate of complications, particularly Clavien-Dindo grades III to V [11]. There was only one case of burst abdomen in the PreM group, but without significant difference. The univariate analysis of risk factors associated with complications demonstrated a higher rate of SSO in obese individuals (OR, 2.6; 95% CI, 1.02–6.90; *p* = 0.04).

**TABLE 2:** Outcomes after midline laparotomy.

N = 121	PreS n = 53	PreM n = 68	*p* value
Hospital stays; median days (IQR)	11 (7.0–11.0)	8 (5.6–16.0)	0.27
Complications; n (%)	44 (83)	56 (87.4)	0.56
No Complications	9 (17)	12 (17.6)	0.91
Clavien-Dindo I	8 (15.1)	14 (20.6)	0.91
Clavien-Dindo II	16 (30.2)	21 (30.9)	0.91
Clavien-Dindo III	11 (20.7)	11 (16.2)	0.91
Clavien-Dindo IV	5 (9.4)	7 (10.3)	0.91
Clavien-Dindo V	4 (7.5)	3 (4.4)	0.91
Reintervention	2 (3.8)	7 (10.3)	0.16
SSO; n (%)	17 (30.2)	16 (23.2)	0.27
Seroma	7 (13.2)	10 (14.7)	0.56
SSI	6 (11.3)	4 (5.9)	0.22
Haematoma	1 (1.9)	5 (7.4)	0.17
Cutaneous dehiscence	3 (5.7)	1 (1.5)	0.22
Evisceration	0	1 (1.5)	0.56
Incisional Hernia; n (%)	2 (3.8)	13(19.1)	0.01
Incisional hernia repair; n (%)	0	4 (30.7)	0.09

IQR, interquartile range; SSO, surgical site occurrence; SSI, surgical site infection.

**FIGURE 2:**
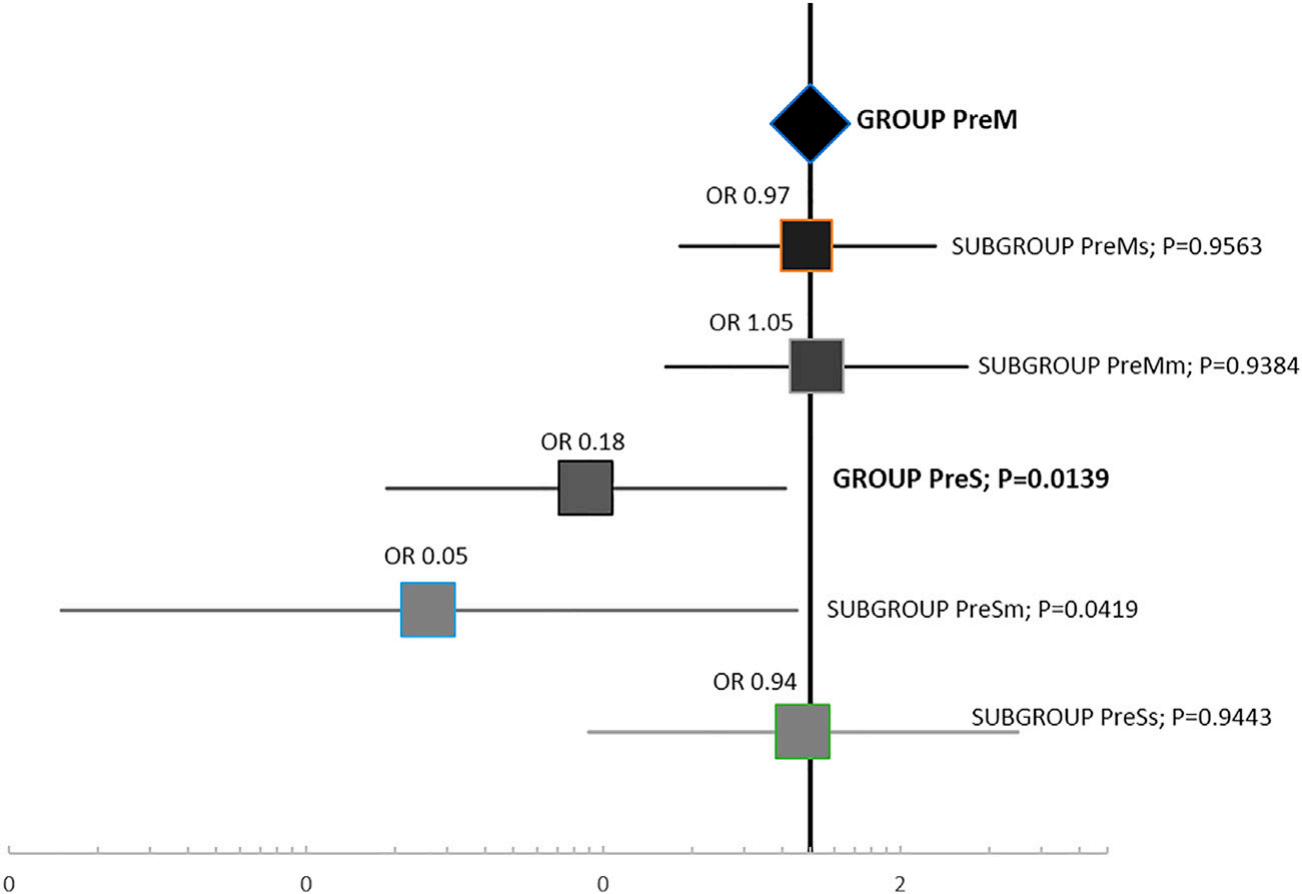
Comparative risk of incisional hernia incidence between groups and subgroups.

A total of 107 patients (88.4%) completed a minimum 6-month follow-up (15.7 ± 13.69 months), and 15 patients were diagnosed with IH (14.4%). The PreM group showed a significantly higher incidence of IH (OR, 1.21; 95% CI, 1.05–1.39; *p* = 0.015) (Figure 2) Subgroup analysis of IH incidence showed the absence of IH in the PreSm subgroup, whereas all of the other subgroups had an incidence of almost 20% (Table 3). Subgroups analysis of comparative IH risk is represented in Figure 2 taking as a reference PreM group with related risk OR subgroups IH risk.

**TABLE 3:** Subgroups long-term incisional hernia incidence. (1year follow-up).

Previous mesh reinforcement n (%)	Prophylactic mesh use n (%)	IH; n (%)
NO; 53 (preS group)	YES; 42 (34)	0 (0)
	NO; 11 (9)	2 (18.2)
YES; 68 (preM group)	YES; 25 (20.6)	5 (20)
	NO; 43 (35.5)	8 (18.6)

**FIGURE 3:**
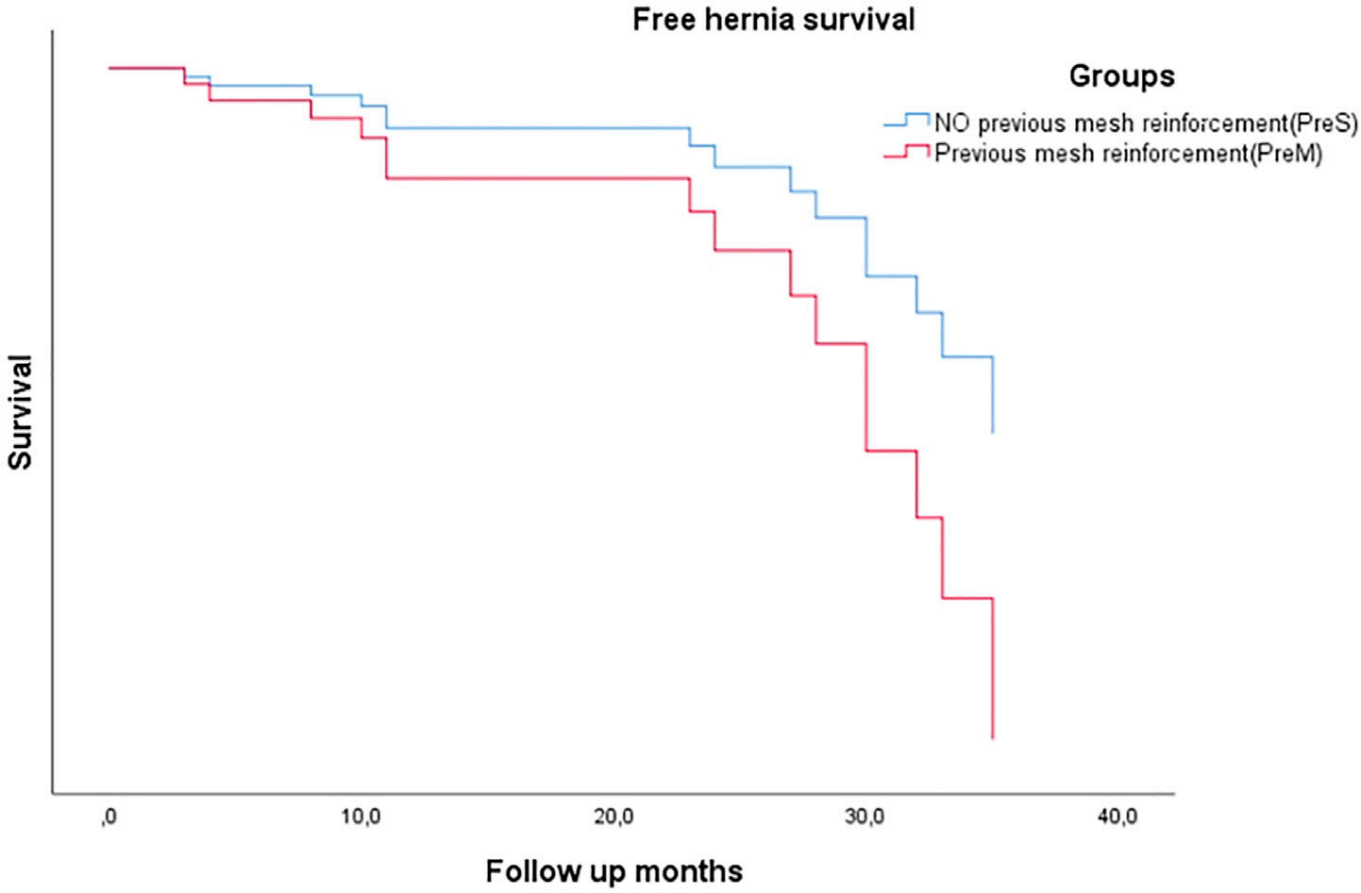
Free hernia survival comparing previous mesh or non-reinforcement groups.

In the analysis of technical factors related to IH, slowly absorbable suture (OR, 0.74; 95% CI, 0.60–0.91; *p* = 0.001), USP 2/0 suture (OR, 0.31; 95% CI, 0.10–0.94; *p* = 0.033), and *SB* technique (OR, 0.81; 95% CI, 0.72–0.90; *p* = 0.011) were associated with a lower IH incidence; however, none of them emerged as a risk factor in a multivariate. Cox analysis of hernia-free survival time comparing PreS and PreM groups is shown in Figure 3.

## Discussion

To our knowledge, the present study is the first analysis of the outcomes of closure of iterative midline laparotomy in patients with a previous onlay mesh reinforcement. This situation probably will be more common in the future due to the suggestions to use a prophylactic mesh in high-risk patients [12–14], but there is limited data on how to act surgically.

Our results showed that, in general, closure of the abdominal wall including the mesh had the same risk of IH as the closure of an IL without mesh, also confirming IL’s high risk of any type of complications as suggested by previous studies [1].

In the subgroup analysis, there was a clear protective effect in patients who received a mesh over previously non-reinforced abdominal wall (subgroup PreSm), while the patients in the remaining subgroups had a similar risk for developing IH. It is interesting to note that closing a previous reinforced abdominal wall showed the same rate of IH, irrespective of the method used (new mesh or suturing previous mesh). It seems that when the mesh is opened, it loses its protective role. A new question arises as to why a new mesh is not effective enough in these patients? This could be explained by its position in the abdominal wall. In our series, all of the prostheses were placed onlay [15]. We hypothesize that better results would have been obtained if the new prophylactic mesh had been placed in other abdominal wall virgin layer, such as retromuscular or intraperitoneal, as has been shown in other studies [16].

A detailed analysis by the suture technique showed that the “*small bites*” technique was associated with lower frequency of IH in the whole population. In patients with any other closure technique, the PreSm patients had better results (0 IH), while the worst results were observed in both PreMm and PreMs subgroups (33% and 53.3% of IH, respectively). In view of these results, we can not say “*small bites”* is a better technique for closure of IL, as has been suggested by previous research [3]. The effect of “*small bites”* for closure of an IL when a mesh is already present deserves a randomised study design.

Traditionally, it has been argued that closing a mesh must be done with a suture made of the same material as the mesh. This would mean using a nonabsorbable thread in most cases. In the PreM group, nonabsorbable suture was associated with higher incidence of IH (27.9% *vs.* 4.3%; *p* = 0.02).

### Strengths and Limitations of the Study

One strong point of our study is prospective and registered data collection. Given its implementation technique model, our study showed results in a real scenario with real or casual problems. Limitations of study are as follows: This was not a randomised study, which could have led to bias in patient’s selection or treatment. As another weak point, given that the protocol recommendation depends on the final decision of surgeon in charge, there is procedural diversity, which is more complicated to analyse. Non blinded follow up and a short median study’s period of follow up could also produce an incisional hernia detection bias. Finally, the small size of the sample, even minimized particularly in subgroup analyses, reduced the statistical power of the study to detect differences.

## Conclusion

In conclusion, IL has a high percentage of complications and IH. In case of IL without previous reinforcement, a mesh can help to reduce IH. Opening a mesh leads to loss of its protective effect, irrespective of the technique of closure. Our data cannot give clear support to any particular technique to close an IL with previous mesh. Further studies on this issue are needed.

## Data Availability

The raw data supporting the conclusions of this article will be made available by the authors, without undue reservation.
